# Comparative Analysis of Work Function Measurement Techniques Based on Scanning Probe Microscopy

**DOI:** 10.1002/adma.74141

**Published:** 2026-07-18

**Authors:** Daryoush Nosraty Alamdary, Matthias Bode, Artem Odobesko

**Affiliations:** ^1^ Physikalisches Institut, Experimentelle Physik II Universität Würzburg Würzburg Germany

**Keywords:** extrapolation, Kelvin probe force microscope, microscopy, scanning capacitance microscopy, scanning probe microscopy, spectroscopy, superconductivity, work function

## Abstract

In this study, we compare three scanning probe techniques—I(z) spectroscopy, field‐emission resonances (FER), and Kelvin probe force spectroscopy (KPFS)—for quantitative local work function measurements. Using Ag(111) as a reference and applying the methods to Ir(111) and Au(111), we find a strong method dependence. I(z) spectroscopy shows substantial uncertainty due to broad data dispersion and is reliable only on flat surfaces. We show that the triangular‐barrier FER extrapolation method lacks robustness, whereas numerical FER analysis based on an image potential‐corrected Schrödinger model yields consistent values. KPFS provides the most direct and reliable work function determination once the tip is calibrated. Applying these methods to superconducting Nb, we extract the work functions of (111) and (110) surfaces and its distinct oxygen‐reconstructed phases.

## Graphical abstract

Non‐contact scanning probe microscopy visualizes the surface topography based on surface electronic properties. The tunneling current, which defines the geometry of the tunnel–junction, has to overcome the effective surface work function barrier to reach the probe from the surface. This barrier can be measured, using specific techniques, such as vibrations of a resonant cantilever, or coupling to quantum confined states.

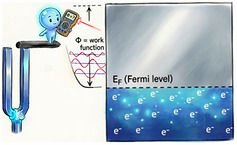

## 1 Introduction

The work function ϕ, defined as the difference between the electrostatic potential of the vacuum and the Fermi level of a material, $\phi =-e\Phi_{\mathrm{vac}}-E_{F}$ [1, 2], is a physical property highly relevant for the functionality of numerous solid‐state devices. It not only determines the minimum energy necessary to set an electron free from a surface into the vacuum [3, 4], but also influences, for example, the electrochemical activity in catalysis [5], the Dirac point position in graphene‐like materials [6], or the band bending of semiconductor heterojunctions [7].

Various techniques have been developed to measure the work function (WF) of materials, including thermionic emission (TE) [2], field emission (FE) [8], the photoemission of adsorbed xenon (PAX) [9, 10], and various methods based on the photoelectric effect (PE) [11] such as photoelectron spectroscopy [12], two‐photon photoemission spectroscopy of image potential states [10], and laser photoemission [13]. While each of these methods has certain strength, they also suffer from specific limitations. For example, TE requires high thermal stability, FE demands tip‐shaped samples, and the PE includes band gap contributions for semiconductors [12]. A more fundamental limitation of these techniques is their inherent spatial averaging, which renders them largely insensitive to local variations which may be caused, for example, by changes in the step edge density [14, 15, 16] or the local adsorption of impurities [17, 18, 19].

Scanning tunneling (STM) and atomic force microscopy (AFM), by contrast, provide direct access to local variations in the electronic structure and surface topography down to the atomic scale. Several STM‐based techniques have been established for nanoscale WF characterization: (i) The I(z) method, based on the exponential decay of tunneling current I with increasing tip–sample separation z [20], provides an estimate of the effective surface barrier, including the WF contribution [21, 22]. (ii) Field emission resonances (FER) observed when the applied bias exceeds the tip's work function yield discrete peaks in the tunneling current whose energy positions reflect the local WF through resonant coupling to junction‐confined states [23, 24, 25, 26]. (iii) Kelvin probe force microscopy and spectroscopy (KPFM/KPFS), implemented in noncontact AFM, map the local contact potential difference with nanometer resolution [27, 28].

Despite the sensitivity of these techniques, a systematic comparison addressing their methodological and practical strength, limitations, and weaknesses remains lacking. Especially, field enhancement effects in FER [29], tip‐induced perturbations in KPFM, and the approximate nature of I(z) spectroscopy introduce uncertainties that complicate quantitative interpretation. A critical evaluation of these methods is therefore essential for establishing reliable local WF measurements at the atomic scale.

In this study, we systematically compare three prominent SPM‐based techniques—I(z) spectroscopy, FER, and KPFS—for quantitative local work function measurements. Using Ag(111) as a reference and the well‐defined noble‐metal surfaces Ir(111) and Au(111) as benchmarks, we assess the accuracy, reproducibility, and junction sensitivity of each method. Beyond this comparison, the present work establishes a practical framework for quantitative nanoscale work‐function determination on metallic and superconducting surfaces with nontrivial local morphology and chemistry. This is demonstrated for clean Nb(111), clean Nb(110), and oxygen‐reconstructed Nb(110), where method‐dependent offsets and spectral artifacts must be carefully disentangled.

## 2 Methods

All measurements were performed using a commercial low‐temperature scanning probe microscope (LT‐SPM) operating under ultrahigh vacuum (UHV) conditions (base pressure $p\leq 1\times 10^{-10}\;\mathrm{mbar}$) and at a sample and tip temperature of 5 K. Sample preparation is carried out in a dedicated UHV chamber, equipped with standard sputtering and annealing facilities, including a home‐built electron‐beam heater and cold field evaporation for qPlus‐sensor tip treatment [30].

Ag(111) and Au(111) substrates were prepared using standard sputtering and low‐temperature annealing cycles ($T_{\mathrm{ann}}<400^{\circ }C$). Ir(111) surface preparation requires a sequence of sputtering and high‐temperature annealing cycles at $T_{\mathrm{ann}}=1100^{\circ }C$ using an electron beam heater. Extended annealing (up to 2 min) was necessary to obtain atomically clean and flat surfaces. Preparation of Nb surfaces involves high‐temperature flashes [31]. Clean Nb(110) is obtained through multiple high‐temperature flashes up to $T_{\mathrm{fl}}=2400^{\circ }C$, that is, just below the melting point. Prior to achieving a clean surface, Nb(110) undergoes the sequential formation of oxide reconstructions. At intermediate flashing temperatures ($T_{\mathrm{fl}}\approx 1700^{\circ }C$), an oxygen‐induced reconstruction ${\mathrm{NbO}}_{x}$ phase i with linearly arranged oxygen mini‐chains appears. Flashing above $2000^{\circ }C$ leads to a short range‐ordered ${\mathrm{NbO}}_{x}$ phase ii. In contrast, Nb(111) could not be prepared atomically flat under these conditions but exhibited three‐dimensional faceting into {011} and {211} pyramidal structures, consistent with earlier results [32].

STM tips were electrochemically etched from a 375μm tungsten wire in NaOH solution and subsequently cleaned and sharpened by field‐assisted poking and pulsing on Ag(111), yielding atomically sharp tips with high field enhancement. For AFM and KPFM measurements, qPlus M4 tuning fork sensors were used as described in Ref. [33]. The sensor was equipped with a 50μm tungsten tip and exhibited an initial resonance frequency $f_{0}=79$ kHz and a spring constant k=1850 N/m [34]). The tip was conditioned by field evaporation at 3 kV for 20 min. After mounting, the resonance frequency was reduced to $f_{0}=41.5$ kHz with a quality factor of approximately $1.7\times 10^{5}$.

In order to ensure reliable and reproducible results, all measurements were initialized on Ag(111), which served as the reference surface for tip preparation and for the pre‐characterization of both STM and AFM tips with respect to their sharpness and work function. The initial conditioning on Ag(111) consisted of measuring the Ag(111) surface state and verifying the lateral resolution by imaging step edges. Ag(111) also served for repeated recalibration of the tip work function during the measurements, especially after noticeable spectral changes. For obtaining sharp STM tips, we employed the tip‐shaper module of the Nanonis software, which applies a programmed sequence of deep poking, bias pulsing, and retraction. For quantitative FER analysis, spectra were recorded on terraces sufficiently large to avoid direct step‐edge effects, as nearby steps can alter the local potential and electric‐field distribution in the junction.

## 3 Theoretical Background

**FIGURE 1:**
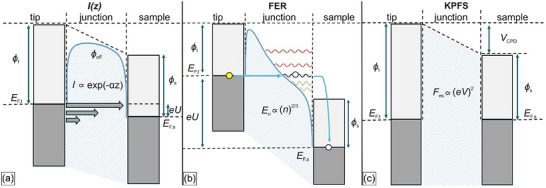
Schematic potential diagrams of (a) I(z) spectroscopy across an effective barrier, (b) the quantum well states in field emission resonances (FER), and (c) the contact potential difference (CPD) in Kelvin probe force spectroscopy (KPFS).

The most prominent method to determine the barrier height, following the early works of Binnig et al. [20] and Tersoff and Hamann [35], is I(z) spectroscopy. The bias dependence of the tunneling current defines three regimes: (i) low bias, (ii) intermediate bias, and (iii) high bias [36]. At low bias the effective barrier is nearly bias‐independent, whereas at high bias it approaches a Nordheim–Fowler form [37]. In the intermediate case (ii) (Figure 1a), the effective barrier height is $\phi_{\mathrm{avg}}=\left(\phi_{s}+\phi_{t}-\left|eU\right|\right)/2$, with $\phi_{s/t}$ being the sample/tip work functions, respectively. From the exponential decay of the tunneling current upon increasing the tip–sample distance z, the sum $\phi_{s}+\phi_{t}$ can be extracted [20, 21, 22, 38]

(1) $$\phi_{\mathrm{avg}}=\frac{\hbar^{2}}{8m}{\left(\frac{d\ln I}{dz}\right)}^{2},$$

where m is the electron effective mass. Experimentally, the current is stabilized at a chosen setpoint, the feedback is opened, and I(z) is recorded over a fixed range.

At sufficiently large positive bias, the junction enters field‐emission regime [37, 39]. Gundlach showed [40] that the tunneling through a trapezoidal potential leads to oscillation of the tunneling current with increasing bias. The corresponding peak energies reflect the quantum well states formed in the trapezoidal potential and are known as FER (Figure 1b). Commonly used approximation of replacing the trapezoidal barrier by a triangular one yields the eigenenergies from the solution of one‐dimensional Schrödinger equation

(2)

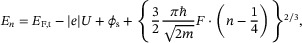

where F is the local field gradient [41, 42]. Resonant tunneling occurs when $E_{n}=E_{F,t}$, producing a characteristic sequence of peaks observed in the dI/dU spectrum. As can be seen, FER peak positions $U_{n}$ plotted against $n^{2/3}$ remain approximately linear, and extrapolation to n=0 provides a practical estimation of $\phi_{s}$.

In the triangular barrier approximation the junction is assumed to have a constant field gradient $F=(|e|U+\phi_{t}-\phi_{s})/z$. In practice, FER measurements are performed under closed feedback‐loop conditions, resulting in a tip retraction in order to keep the set current, which sequentially changes z. Thus, the effective field gradient does not necessary remain constant. Furthermore, at small z the image potential

(3) $$V\left(z\right)=-\frac{e^{2}}{16\pi \varepsilon_{0}z}$$

becomes comparable [26, 43], producing an additional distortion of the barrier shape. Thus, a reliable determination of the WF requires fitting the FER peak sequence with a numerically solved Schrödinger equation using a modified, realistic junction potential rather than the ideal triangular approximation (see Appendix D, Figure S7).

In contrast to I(z) and FER spectroscopy, KPFS probes the electrostatic interaction between an oscillating cantilever and the surface. This interaction results in a frequency shift [44]

(4) $$\Delta f\left(z\right)=\frac{f_{0}}{2k}\frac{\partial F_{\mathrm{el}}\left(z,U\right)}{\partial z},$$

where $f_{0}$ is the eigenfrequency of the cantilever, k is the cantilever stiffness, and $\partial F_{\mathrm{el}}/\partial z$ is the tip–sample distance z‐dependent force gradient [45]. The frequency shift is detected in the phase‐locked loop.

In analogy to a plate capacitor with capacitance C, a tip–sample force arises from the electrostatic interaction, which scales quadratically with the potential difference U [45]

(5) $$F_{\mathrm{el}}=\frac{1}{2}\frac{\partial C}{\partial z}{\left(U_{\mathrm{el}}\right)}^{2}.$$

The electrostatic potential, hereby, is $U_{\mathrm{el}}=U-\frac{\Delta \phi }{e}$, where $\Delta \phi =\phi_{t}-\phi_{s}$ is the work function difference or contact potential difference (Δϕ=−CPD), see Figure 1c. The resulting quadratic dependence exhibits a minimum when the applied bias compensates the CPD. Thus, with a characterized tip, the sample work function $\phi_{s}$ follows directly from the bias at which the frequency‐shift parabola reaches its minimum.

## 4 Results and Discussions

### 4.1 Reference Measurements on Ag(111)

**FIGURE 2:**
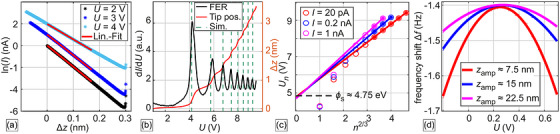
Spectroscopic reference data measured on clean Ag(111). (a) I(z) spectra; setpoint parameters: I=200 pA, bias voltage U=2, 3, and 4 V. (b) Field emission resonances (FER) observed in the dI/dU signal (black), tip retraction (red), and the simulated FER peak positions (dashed green); setpoint parameters: I=200 pA. (c) Extrapolation of the FER peak positions $U_{n}\left(n^{2/3}\right)$ obtained from FER data in (b) and measured at I=20 pA, 200 pA, and 1 nA. (d) KPFS spectra measured at oscillation amplitudes $z_{\mathrm{amp}}=7.5$, 15, and 22.5 nm.

Figure 2 summarizes reference measurements on clean Ag(111): (a) I(z) spectroscopy, (b,c) FER, and (d) KPFS. The I(z) spectra were taken at three distinct bias voltages of U=2, 3, and 4 V. On a semi‐logarithmic scale they show a linear decay, as expected from Equation (1), with the slope reflecting the average barrier height $\phi_{\mathrm{avg}}$. Using the known work function of Ag(111) ($\phi_{s}\approx 4.64$ eV) [46], the extracted tip work functions vary substantially, yielding $\phi_{t}\approx 4.6;5.2;3.2$ eV for U=2;3;4 V, respectively. This strong bias dependence illustrates the limited reliability of the I(z) method for a quantitative determination of the work function.

Figure 2b shows FER measurements. The dI/dU signal (black line) exhibits a series of peaks that move closer together and decrease in amplitude with increasing bias. The tip retraction Δz (red line) under closed‐loop, constant‐current conditions shows a staircase‐like behavior: each FER induces a sharp upward step due to the increased tunneling current and the corresponding rapid feedback‐induced retraction, whereas the segments between resonances display a smooth, monotonic withdrawal of the tip. The extracted tip retraction $\Delta z(U_{n})$ is used in numerical FER analysis to model the junction width. The peak positions are fitted by solving the Schrödinger equation for the corresponding barrier profile while varying the unknown surface/tip work function to minimize the deviation from experiment (see Supporting Information, Appendix D for details). The best fit, shown by the green dashed line, corresponds to a tip $\phi_{t}=4.5\;\mathrm{eV}$, consistent with the typical work function of tungsten [46].

Figure 2c shows the extracted FER peak voltages $U_{n}$ plotted versus $n^{2/3}$ for set currents of 20 pA, 0.2 nA, and 1 nA. Within the triangular‐barrier approximation, a linear extrapolation to n=0 yields the sample work function and, according to Equation (2), does not require knowledge of the tip work function. To minimize the influence of the image potential‐induced deviations of the barrier, the lowest‐order resonances must be excluded from the analysis. Restricting the fit to n>5 provides consistent values, giving a Ag(111) work function $\phi_{s}=\left(4.75\pm 0.1\right)\;\mathrm{eV}$.

Figure 2d shows the frequency shift Δf(U) in KPFS, recorded at oscillation amplitudes of 7.5, 15, and 22.5 nm. Each curve exhibits a parabolic dependence with decreasing curvature at larger amplitudes. The parabolas share a common minimum at U=(0.25±0.05) V, corresponding to the contact potential difference. Knowing the $\phi_{s}$ of Ag(111), we obtain a tip work function of $\phi_{t}\approx 4.4$ eV, in good agreement with FER simulations.

These measurements on Ag(111) therefore provide a reliable characterization of the tip work function. The calibrated $\phi_{t}$ is then used as an input parameter for determining the work functions of other surfaces, where $\phi_{s}$ is not known a priori.

### 4.2 Influence of the Tip Sharpness on FER

**FIGURE 3:**
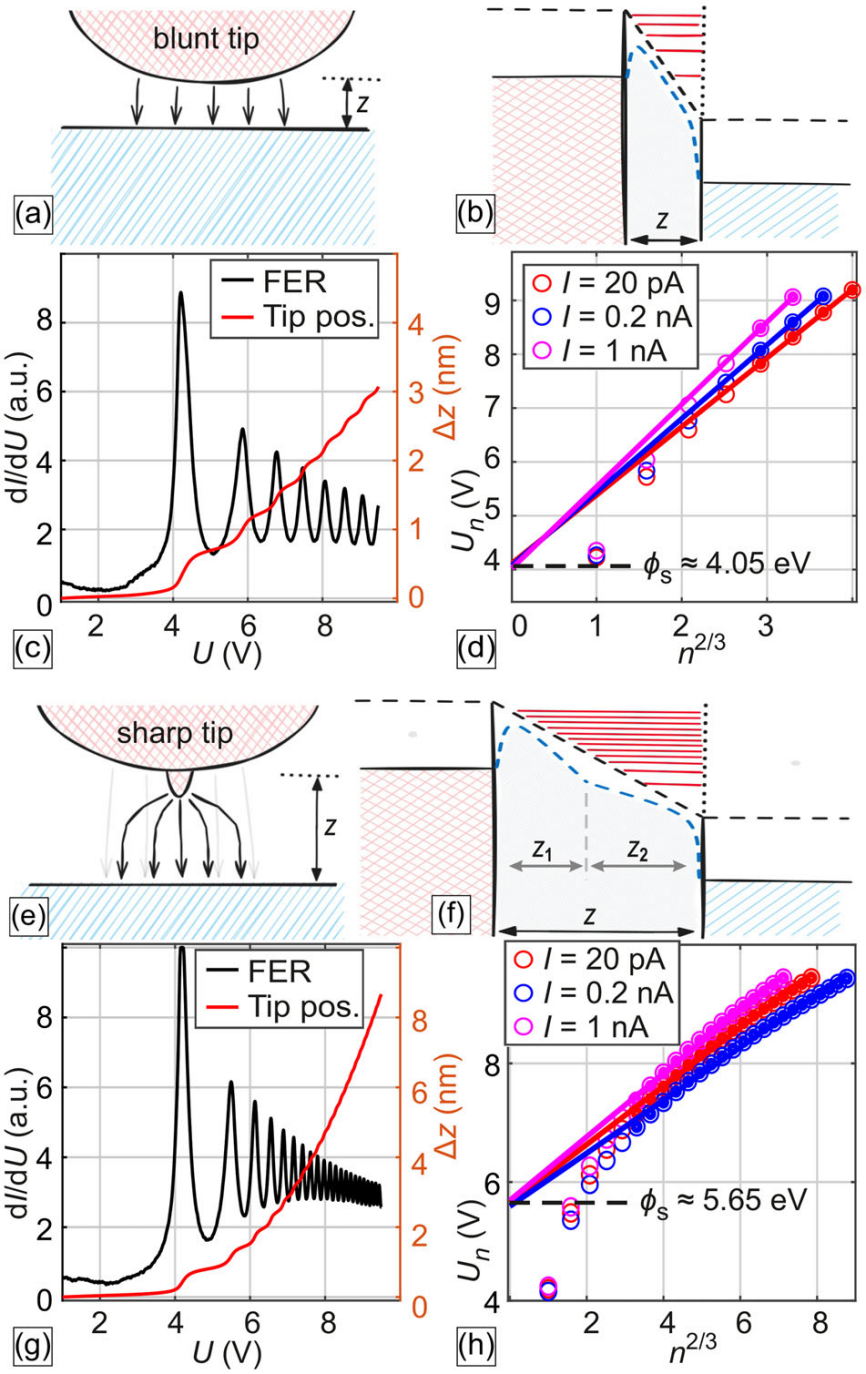
(a) Schematic of the tip–sample geometry and the resulting electric‐field distribution for a blunt STM tip. (b) Corresponding energy diagram at positive sample bias, showing the idealized trapezoidal tunneling barrier (black dashed) and the actual barrier including the image potential (blue dashed), together with the associated FER levels (red lines). (c) FER spectrum (black) and tip retraction Δz (red) measured on Ag(111) with a blunt tip at I=200 pA. (d) Extrapolation of the peak voltages from (c) plotted versus $n^{2/3}$ for set currents of 20 pA, 200 pA, and 1 nA. (e–h) Same as (a–d) for a sharp STM tip. For sharp tips, strong local field enhancement at the apex produces a highly nonuniform barrier with a steep field‐gradient region near tip $z_{1}$ and a much shallower region near sample $z_{2}$.

Variations in the tip apex geometry have a pronounced influence on the number and spacing of FERs, even on the same surface. Figure 3 illustrates two limiting cases: a blunt tip showing only a few resonances [(a),(c)] and a sharp tip producing a dense sequence of peaks [(e),(g)] on Ag(111). In the sense of the electrostatic junction model introduced in Appendix D, a tip is referred to as “blunt” when its apex radius is much larger than the tunneling‐junction width, such that the junction geometry approaches the planar‐capacitor limit with an almost homogeneous electric field. A tip is referred to as “sharp” when its apex radius becomes comparable to the tunneling‐junction width, leading to a strongly inhomogeneous field and local field enhancement at the apex. This field enhancement assists field‐emission tunneling and causes substantially larger retractions during the bias sweep. For instance, Figure 3c,g shows a total retraction of about 3 nm for the blunt tip but more than 8 nm for the sharp one. The larger retraction increases the effective confinement width and yields a larger number of FER states.

In both limits the tunneling barrier deviates substantially from the ideal trapezoidal form assumed in the triangular‐barrier approximation (Equation 2). For blunt tips, the image potential dominates at small tip–sample distances and distorts the barrier near the surface. For sharp tips, the strong local field enhancement produces a steep field gradient near the apex and a much weaker gradient near the surface, resulting in a highly nonuniform potential profile.

These deviations introduce systematic errors in the FER‐based work function extrapolation. Blunt tips provide too few usable resonances once the image‐potential‐affected peaks (typically the first four to six) are removed, which leads to an underestimation of the surface work function, see Figure 3d. Sharp tips with strong field enhancement, on the other hand, effectively split the barrier into two regions, $z_{1}$ and $z_{2}$, with distinct field gradients near the apex and the surface (Figure 3f). This asymmetry causes a systematic overestimation of the work function when the triangular‐barrier model is applied (Figure 3f), as also reported in previous works [25, 29].

These observations suggest that an intermediate tip sharpness is required for reliable FER‐based work‐function extrapolation. The Ag(111) spectra in Figure 2b were obtained with such a tip, with a sufficient number of FERs while deviations from the triangular‐barrier approximation remain limited. For a more accurate and generally applicable analysis the junction potential and the resulting FER spectrum must be modeled explicitly by solving the Schrödinger equation, including the image potential, possible dielectric layers, and the field‐enhancement near the apex. This approach is used for the work‐function determination presented here.

### 4.3 Application to Dense‐Packed Noble‐Metals and Nb Surfaces

To examine the broader applicability of the methods, we extended the measurements to the dense‐packed (111) surfaces of the nobel metals Ir and Au, to the superconducting bcc Nb(111) surface, and to a set of clean and oxidized terminations of Nb(110). The work functions of Ir(111) and Au(111) are well established, and the recommended values from Ref. [46] provide a reliable benchmark for assessing the accuracy of the different techniques. Representative topographies, FER spectra, and qPlus frequency‐shift maps are provided in the Supporting Information.

For all surfaces we applied the same three methods: (i) I(z) spectroscopy, (ii) FER spectroscopy, and (iii) KPFS. The extracted work‐function values are summarized in Table 1.

**TABLE 1:** Summary of work function mean values obtained by I(z) spectroscopy, FER, and KPFS measurements on various surfaces.

Method/Material	I(z)	FER, sharp tip $U_{n}\left(n^{2/3}\right)$ extrap.	FER, sharp tip numerical model	FER, blunt tip numerical model	KPFS	Literature
Ir(111)	5.7±0.2	6.2±0.2	6.1±0.1	6.3±0.1	5.55±0.15	5.75±0.06
Au(111)	6.6±0.9	5.2±0.2	5.4±0.1	5.7±0.2	5.18±0.06	5.33±0.09
Nb(111)	3.6±0.3	5.4/4.6±0.3	4.9/4.7±0.1	4.5/4.4±0.2	4.26/4.21±0.02	3.95±0.09
clean Nb(110)	4.2±0.2	5.3±0.4	5.4±0.1	5.3±0.1	4.96±0.08	4.77±0.05
${\mathrm{NbO}}_{x}$ phase i	3.3±0.5	5.5±0.2	5.1±0.1	5.0±0.1	5.0±0.1	—
${\mathrm{NbO}}_{x}$ phase ii	3.5±0.3	5.4±0.3	5.2±0.2	4.8±0.1	4.78±0.05	—
STM W tip	4.5±0.2	—	4.8±0.1	—	—	4.56±0.03
qPlus W tip	—	—	—	4.6±0.1	4.47±0.08	4.56±0.03

*Note*: All values are given in eV. Error represent the spread deviation. Literature values taken from Ref. [46].

The I(z) spectroscopy occasionally reproduces the expected work function (as on Ir(111)), but generally shows substantial scatter and may deviate strongly from the reference values, as clearly seen for Au(111). Because the analysis relies solely on fitting an exponential decay of the tunneling current—an outcome that also arises for many nonideal barrier shapes—the method offers no internal indication whether the extracted effective barrier height is reliable. This limitation has also been noted in previous comparative studies [47, 48].

For the FER‐based WF determination, three columns are shown. The first corresponds to the linear extrapolation of $U_{n}\left(n^{2/3}\right)$, applicable for a data with sufficiently large number of resonances (n≳8), that is, for sharp tips. Blunt tips, which produce only a few peaks—on surfaces with a high work function such as Ir(111) and Au(111) typically 3 or 4—are unsuitable because the lowest FERs are strongly modified by the image potential and therefore do not follow the triangular‐barrier model. Sharp tips generate many resonances, however, including high‐order states in the extrapolation leads to a systematic overestimation of the work function. For large tip–sample separations the resonances no longer form within a common triangular well: each high‐n level originates from a distinct, distance‐dependent potential whose shape becomes increasingly curved due to changes in the electrostatic field across the junction. This evolution results in a gradual reduction of the slope in the $U_{n}$ versus ${\left(n\right)}^{2/3}$ dependence, thereby invalidating the triangular‐barrier approximation. Restricting the fit to intermediate resonances (2<n<6), as proposed in Ref. [49], introduces noticeable uncertainty and remains highly sensitive to the specific set of FER peaks included. In our analysis, we exclude the first 4 and 5 FERs to mitigate image‐potential distortions; while this yields reasonable values, the triangular‐barrier approximation still lacks robustness for quantitative work‐function determination because the outcome depends sensitively on the chosen peak range.

A more reliable approach that avoids selective peak inclusion is the numerical modeling of the FER spectrum, columns 4 and 5 in Table. 1. In this analysis, all observed resonances are fitted simultaneously using a junction potential reconstructed from the measured tip retraction Δz(U), which also explicitly incorporates the image potential and the field‐enhancement effects characteristic of both blunt and sharp tips. The work function is obtained by fitting the experimentally obtained FER peak positions in numerical calculations. For high‐work‐function Au(111) and Ir(111) surfaces, the numerical modeling tends to give slightly larger $\phi_{s}$ values than those commonly reported in the literature. Nevertheless, the convergence of the simulations is very robust: varying the initial parameters of the junction model consistently reproduces the work‐function values within the error bar listed in Table 1.

KPFS provides the most robust and internally consistent work‐function values among the three methods applied. Once the tip work function is calibrated on Ag(111), the contact potential difference is obtained directly from the minimum of the Δf(U) parabola, without requiring any assumptions about the junction geometry or field distribution. For both Au(111) and Ir(111), the KPFS values in Table 1 lie close to the recommended reference data, with smaller scatter than observed in the I(z) or FER analyses. The method is therefore experimentally straightforward and quantitatively reliable.

We next applied the three methods to the strongly corrugated Nb(111) surface, which faceted after high‐temperature preparation into three‐dimensional {211} pyramids of maximum ∼ 1 nm height, separated by flat triangular valleys exhibiting a (2×2) reconstruction (see Appendix C, Figure S5). Both KPFS and FER measurements show a notable site dependence. With sharp tips, spectra on the pyramid peaks yield an apparent work function of ∼4.9 eV, whereas measurements in the valleys give ∼4.7 eV; the triangular‐barrier extrapolation $U_{n}\left(n^{2/3}\right)$ amplifies this contrast to 5.4 eV versus 4.6 eV. In contrast, FER acquired with blunt tips shows only a weak peak–valley difference (4.5 eV on peaks and 4.4 eV in valleys). KPFS performed at small oscillation amplitudes (<1.5 nm) yields 4.26 ± 0.02 eV on peaks and 4.21 ± 0.02 eV in valleys. At larger amplitudes (≳7 nm), this residual contrast disappears entirely, and both sites converge to a common value of 4.30±0.02 eV. In KPFS, the bias dependence is governed by the electrostatic force gradient (Equation 4) and therefore by the effective capacitance derivative of the full tip–sample geometry. Thus, on corrugated surfaces the measured CPD becomes a geometry‐dependent weighted average, and larger oscillation amplitudes increase the lateral averaging while at the same time suppressing the apparent peak–valley contrast. These observations demonstrate that for sharp‐tips the FER method is sensitive to the local junction geometry, whereas KPFS reflects a geometry‐dependent electrostatic averaging of the local CPD. Consequently, blunt‐tip FER and large‐amplitude KPFS yield a laterally averaged work‐function estimate on corrugated surfaces.

**FIGURE 4:**
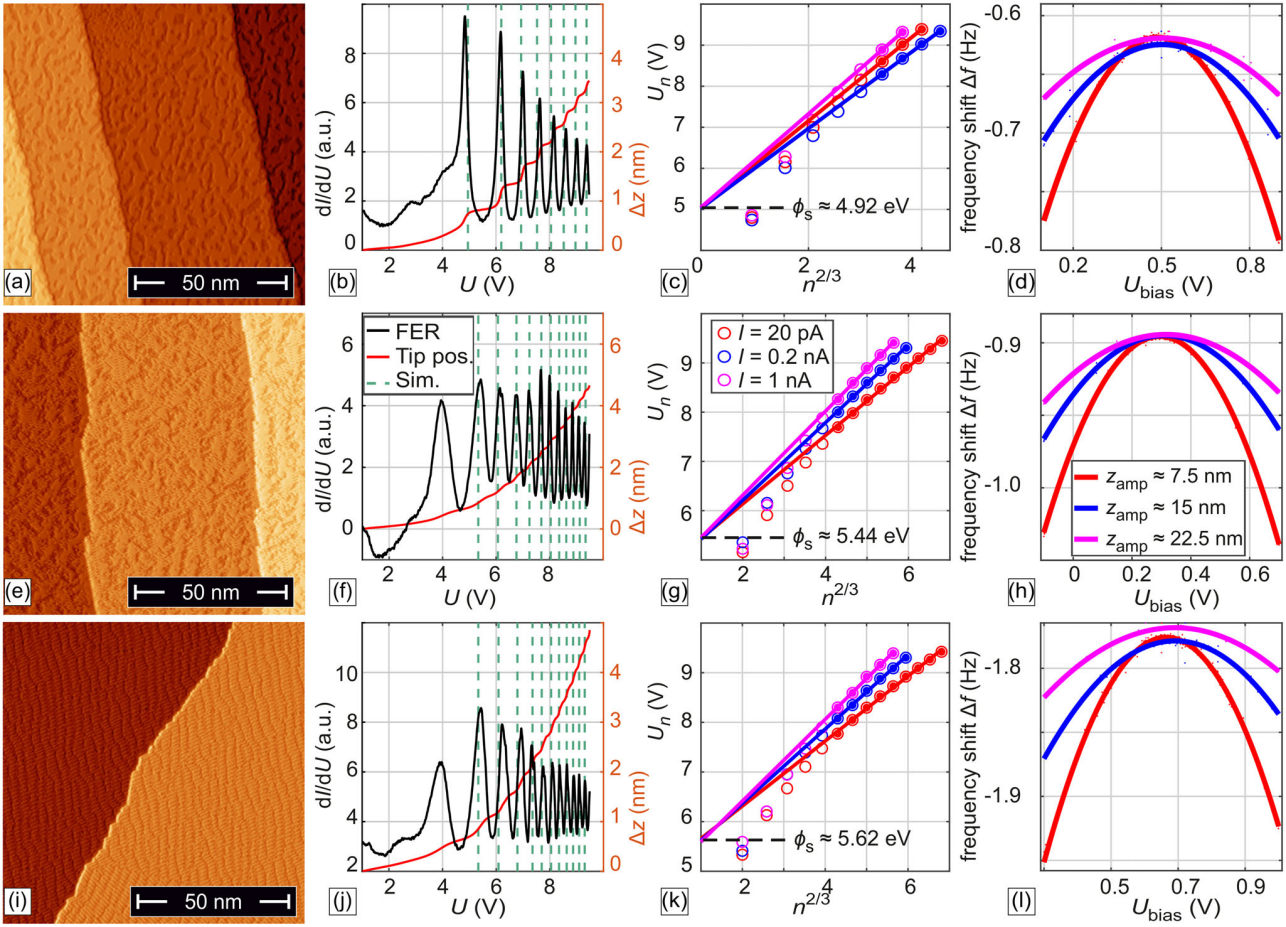
(a) The STM topographic image of a Nb(110) surface containing occasional ${\mathrm{NbO}}_{x}$ patches. (b) FER spectrum measured on clean Nb(110) with a sharp tip; the green dashed line shows the simulated FER spectrum providing the best fit to the experimental data. (c) Extrapolation of the FER peak positions $U_{n}$ plotted against $n^{2/3}$ for three current setpoints (I=20 pA, 200 pA, and 1 nA); (d) KPFS spectra acquired above clean Nb(110) for oscillation amplitudes $z_{\mathrm{amp}}=7.5$, 15, and 22.5 nm. (e–h) Same set of measurements for the ${\mathrm{NbO}}_{x}$ phase ii surface; (i–l) Same measurements for the ${\mathrm{NbO}}_{x}$ phase i surface. Imaging parameters: (a) U=−1 V, I=200 pA; (e) U=−200 mV, I=200 pA; (i) U=−200 mV, I=200 p.

A reliable work‐function determination for Nb(110) requires an oxygen‐free Nb(110) surface, which is difficult to prepare [31]. The available literature values remain limited [46]. Figure 4 summarizes the STM topographies, *I*(z), FER, and KPFS data obtained on partially clean Nb(110) and on two oxygen‐reconstructed terminations, denoted as ${\mathrm{NbO}}_{x}$ phase i and phase ii. The topographic images in panels (a), (e), and (i) were acquired in constant‐current STM mode using the qPlus M4 sensor; the corresponding frequency‐shift images of the same surface areas are shown in Figure S8. Figure 4 summarizes the *I*(z), FER, and KPFS data obtained on partially clean Nb(110) and on two oxygen‐reconstructed terminations, denoted as ${\mathrm{NbO}}_{x}$ phase i and phase ii.

KPFS yields a work function for clean Nb(110) that is about 0.2 eV above the recommended reference value, whereas the FER analysis gives values roughly 0.5 eV higher. Given that FER fit systematically produces larger values than KPFS throughout our study, the work function of clean Nb(110) is expected in the range 5.0–5.2 eV. According to KPFS, ${\mathrm{NbO}}_{x}$ phase i shows a similar value, whereas phase ii is lower by ∼0.2 eV. As can be seen from Figure 4f–g, the FER spectra measured on oxygen‐reconstructed surfaces exhibit an additional resonance near 4 eV, which is absent for clean Nb(110). Including this peak in the FER simulations produces an unrealistically low work function which is ≈0.7−1.0 eV below that of clean Nb(110), a result at odds with KPFS data. A similar feature at ≈4 eV has been observed in inverse photoemission on nominally “clean” Nb(110) and attributed to an unoccupied Nb d‐band [50]. It is now understood that such the preparation conditions reported in Ref. [50] cannot result in clean Nb(110) but leave behind substantial amounts of oxygen on the surface [31]. These oxygen adsorbates strongly hybridize with Nb 4d states and redistributes spectral weight in the occupied and unoccupied LDOS, naturally producing this resonance at 4 eV. Excluding this peak from the FER fit and applying a thin‐dielectric correction (≈200 pm, consistent with an oxygen overlayer; see Supporting Information), restores quantitative agreement between the FER and KPFS data.

As a summary, FER simulations and KPFS represent the two most complementary and internally calibrated approaches. On corrugated surfaces, sharp‐tip FER and low‐oscillation‐amplitude KPFS remain sensitive to local surface morphology and junction geometry, whereas blunt‐tip FER and high‐oscillation‐amplitude KPFS yield a laterally averaged value and deliver a more uniform and consistent work function estimate across a larger area of the surface. KPFS provides work‐function values with small scatter and close to the literature reference when that the tip is accurately calibrated on a well‐known surface such as Ag(111). Numerical FER analysis, in contrast, is markedly more tolerant to uncertainties in the tip work function. Because the junction potential is reconstructed from the experimentally measured tip retraction Δz(U), the tip work function enters mainly as a boundary offset. Moderate variations of $\phi_{t}$ (within ±0.3 eV) shift the overall energy scale but leave the junction field distribution essentially unchanged. Consequently, the simulated FER spectra converge reliably to the same $\phi_{s}$ regardless of the initial choice of $\phi_{t}$.

Systematic measurements reveal that FER spectra taken at low current setpoints (e.g., 20 pA) yield slightly lower work functions than those recorded at higher $I_{\mathrm{set}}$. This trend, observed for both blunt and sharp tips, arises from the reduced electric field when the tip is farther from the surface. Under these conditions the junction approaches a more parallel‐plate geometry. An analogous effect appears in KPFS. At small oscillation amplitudes the sensor probes the strongly curved, high‐field region near the surface, biasing the Δf(U) minimum toward lower voltages. At larger amplitudes (≳10 nm), the electrostatic force is averaged over a wider region of reduced field curvature, yielding a more laterally averaged CPD and thus a more representative surface‐averaged work function.

Overall, both FER and KPFS give the most consistent results under weak‐field conditions, that is, for low $I_{\mathrm{set}}$ for FER, and large oscillation amplitudes for KPFS. FER simulations remain advantageous because they tolerate moderate uncertainties in $\phi_{t}$, although FER‐derived work functions tend to be slightly higher than those obtained by KPFS. The remaining offset between FER and KPFS reflects the fact that the FER analysis relies on a reconstructed junction‐potential model, whose accuracy is limited both when only a small number of resonances is observed and when the fitted field‐enhancement parameter becomes too large, whereas KPFS derives the work function from the electrostatic CPD. In practice, the most consistent FER‐based work‐function estimates are obtained for intermediate junction‐field conditions. Care must also be taken to exclude spectral features originating from the local density of states, such as the first peak on Au(111) or the ∼4 eV oxygen‐related resonance on Nb(110). For these reasons, cross‐validation between KPFS and numerical FER analysis remains essential for an unambiguous determination of surface work functions.

## 5 Conclusions

Our comparison of several scanning probe microscopy‐based approaches demonstrates that quantitative work function determination is strongly method‐dependent. The I(z) technique and the triangular‐barrier FER extrapolation lack robustness, as their results vary with junction conditions and with the number and quality of observable resonances. In contrast, numerical FER analysis based on an image‐potential‐corrected junction yields consistent values with minimal assumptions and remains stable even under moderate uncertainties in the tip work function. Kelvin probe force spectroscopy provides an equally reliable and internally calibrated reference, independent of electronic‐structure details or thin dielectric overlayers. Applying these techniques to Nb surfaces, including previously uncharacterized oxygen reconstructions on Nb(110), we obtain consistent work‐function values and identify spectral features that must be excluded for accurate FER analysis.

## Author Contributions

D.N.A., A.O., and M.B. conceived the experiments. D.N.A. conducted the measurements and analyzed the data. A.O. performed the simulations and analyzed the data. M.B. and A.O. supervised the project. All authors discussed the results. D.N.A. and A.O. wrote the manuscript with input from all authors.

## Conflicts of Interest

The authors declares no conflicts of interest.

## Supporting information

Supporting Information

## Data Availability

The data that support the findings of this study are available on request from the corresponding author. The data are not publicly available due to privacy or ethical restrictions.
