# Supplementary material for: Comparative Analysis of Work Function Measurement Techniques Based on Scanning Probe Microscopy
Source: Adv Mater. 2026 Jul 18;38(45):e74141. doi: 10.1002/adma.74141 (PMC13471934; doi:10.1002/adma.74141)
Supplement: Supplementary file 1 [file ADMA-38-e74141-s001.pdf]

Supplemental Material: Comparative Analysis of Work Function Measurement Techniques Based on Scanning Probe Microscopy

Daryoush Nosraty Alamdary,[1] Matthias Bode,[1] and Artem Odobesko[1]

[1] *Physikalisches Institut, Experimentelle Physik II, Universität Würzburg, Am Hubland, 97074 Würzburg, Germany*

Appendix A: Topographic data

In this section we present experimental results obtained for Ir(111), Au(111) and Nb(111). Together with Ag(111), topographic images of their surface structure are presented in Fig. S1. All images were measured at the same tunneling parameters ($I = 200\,\mathrm{pA}$ and $U = -200\,\mathrm{mV}$) and show a scan frame of $100\,\mathrm{nm} \times 100\,\mathrm{nm}$. The Ag(111) surface of Fig. S1(a) shows three atomically flat terraces separated by two monoatomic step edges. For Ir(111) we find a qualitatively similar surface topography, but detailed comparison reveals several mounds with a typical diameter of a few 10 nm and a height of several 10 pm, see Fig. S1(b). They are caused by subsurface nanocavities caused by argon implantation during the sputtering process [1]. In contrast, the Au(111) surface presented in Fig. S1(c) exhibits a characteristic $\left(22 \times \sqrt{3}\right)$ or so-called herringbone reconstruction caused by a periodic variation of the surface layer's stacking order between hcp and fcc [2]. Finally, the Nb(111) surface displayed in Fig. S1(d) is strongly faceted and consists of sharp {211}-oriented pyramids and valleys. The faceting most likely arises from excessive annealing [3], a treatment that was required to eliminate the highly stable oxygen contamination from the surface.

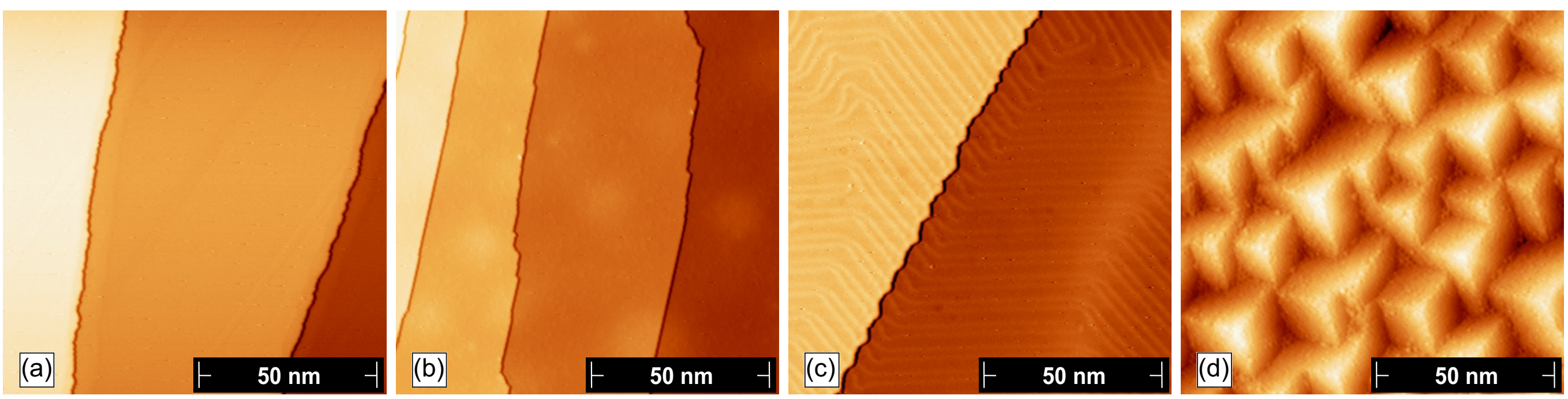

FIG. S1. Topographic images of (a) Ag(111), (b) Ir(111), (c) Au(111), and (d) Nb(111). Imaging parameters: $I = 200\,\mathrm{pA}$ and $U = -200\,\mathrm{mV}$. Each scan frame covers an area of $100\,\mathrm{nm} \times 100\,\mathrm{nm}$.

Appendix B: Spectroscopic data of Ir(111), Au(111), and Nb(111)

In Fig. S2 and Fig. S3 we present the work-function measurements on Ir(111) and Au(111). Each measurement series succeed reference FER and KPFS on Ag(111) to ensure a stable and well-calibrated tip work function. As in the main text, the $I(z)$ method yields values of the work function which strongly deviate from the literature values [4].

FER spectra were analyzed using numerical Schrödinger-based simulations that include the image potential and the experimentally measured tip retraction. For Au(111), the first feature near 3.7 V must be excluded from the FER fit. It corresponds to the onset of the surface-projected bulk conduction band, which appears as a pronounced step in the $\mathrm{d}I/\mathrm{d}U$ signal [5, 6]. Genuine FER peaks emerge at higher voltages.

KPFS provides work-function values in close agreement with the literature and exhibits significantly smaller scatter than either triangular-barrier FER extrapolation or FER simulations (see Tab. ??).

Figure S4 summarizes the $I(z)$, FER, and KPFS measurements obtained on Nb(111). After high-temperature preparation, the surface exhibits pronounced faceting into {211}-oriented pyramids. Work-function values extracted from $I(z)$ and from triangular-barrier FER extrapolation deviate substantially from the recommended reference value. In contrast, FER simulations, as well as KPFS measurements, yield work-function values 0.3-0.4 eV higher than the

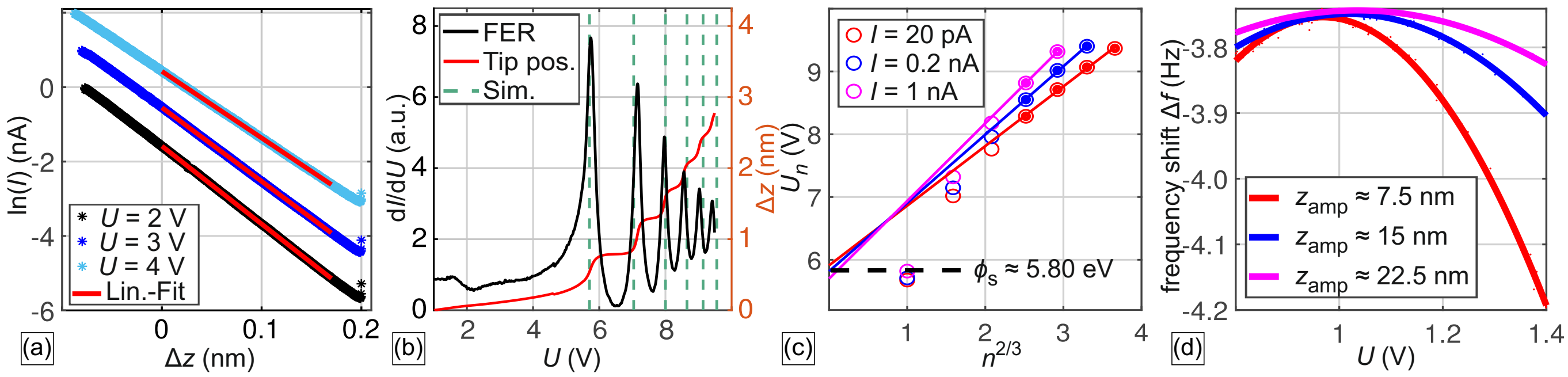

FIG. S2. Spectroscopic data measured on Ir(111). (a) $I(z)$ spectra recorded at setpoint parameters $I = 200\,\mathrm{pA}$ and bias voltages $U = 2$, 3, and 4 V. (b) FER are shown in the $\mathrm{d}I/\mathrm{d}U$ signal (black) together with the corresponding tip retraction (red), measured at $I = 200\,\mathrm{pA}$. The simulated FER are shown in green dashed vertical lines. (c) Extrapolation of the FER peak positions U_n plotted against $n^{2/3}$ for set currents of 20 pA, 200 pA, and 1 nA. (d) KPFS spectra acquired at oscillation amplitudes $z_{\mathrm{amp}} = 7.5$, 15, and 22.5 nm.

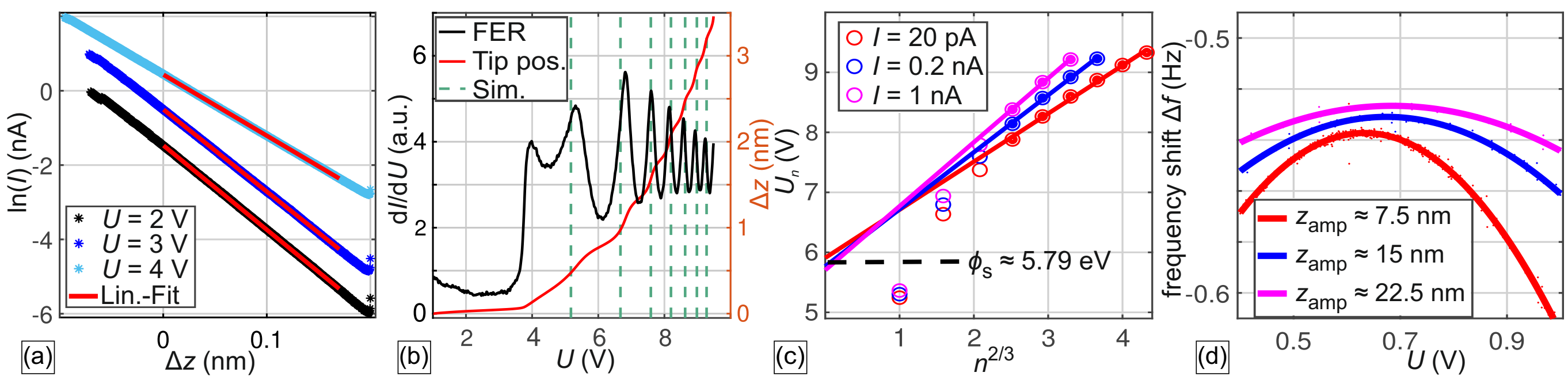

FIG. S3. Same as Fig. S2, but for Au(111).

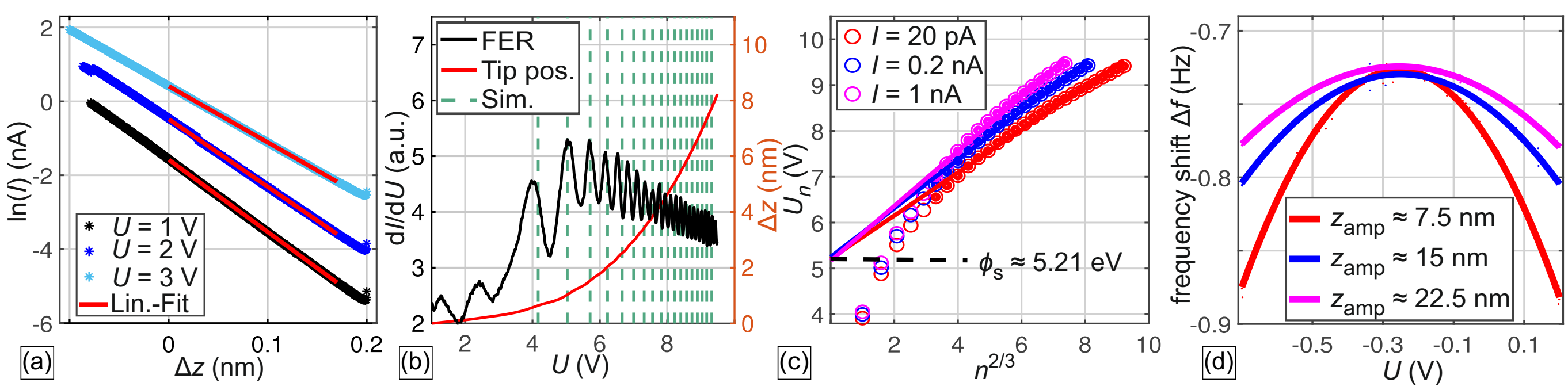

FIG. S4. Same as Figs. S2 and S3, but for Nb(111).

recommended literature values [4]. Data presented in Fig. S4 are acquired at the pyramid peaks. Spatially resolved data illustrating peak-valley site dependence estimations are provided in Sec. C.

Appendix C: Spatially resolved measurements on Nb(111)

Nb(111) develops pronounced faceted pyramids after high-temperature cleaning, resulting in a strongly corrugated surface (Fig. S1d). Additional sputter–anneal cycles create small flat valleys between the pyramids, exhibiting a (2×2) reconstruction (Fig. S5a).

FER measurements reveal a clear site dependence: spectra recorded on the pyramid peaks yield higher apparent work-function values than those measured in the valleys. FER extrapolation for the data measured at peak and valley sites differ significantly, 5.54 eV on the peak and 4.88 eV in the valley. Numerical FER simulations for sharp

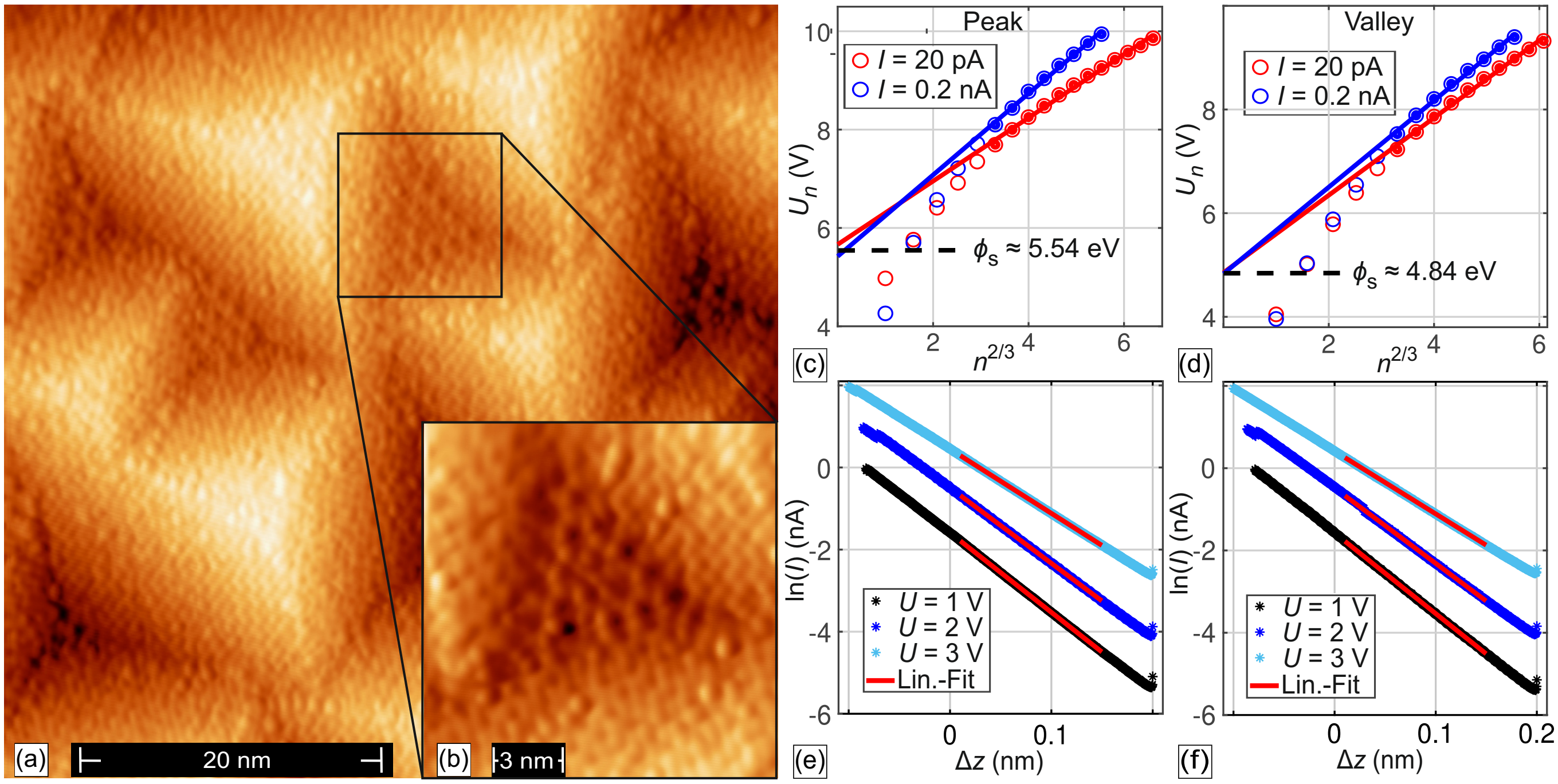

FIG. S5. (a) Overview topographic STM image of the surface of a Nb(111) sample obtained by numerous cycles of sputtering and annealing at reduced temperature ($T_{\mathrm{ann}} \approx 1700\,\mathrm{K}$). (b) Zoom-in of the (2×2)-reconstructed flat valley located in between three pyramids . (c,d) FER measurements performed in peaks and flat valley of the Nb(111) surface, respectively. The data are fit by a simulation model described in Sec. D. (e,f) $I(z)$ spectra recorded at setpoint parameters $I = 200$ pA, and bias voltages $U = 1, 2$, and 3 V, for peak and valley respectively. Imaging parameters: (a,b) $I = 200\,\mathrm{pA}$, $U = -200\,\mathrm{mV}$; scan sizes: (a) $50\,\mathrm{nm} \times 50\,\mathrm{nm}$, (b) $15\,\mathrm{nm} \times 15\,\mathrm{nm}$.

tips give $\phi_{\mathrm{s}} \approx 4.9\,\mathrm{eV}$ on peaks and $\approx 4.7\,\mathrm{eV}$ in valleys. For the FER with the blunt tips this difference is less pronounced, and even less is the difference in KPFS measured at low oscillation amplitudes. see Table 1. For the $I(z)$ spectroscopy, measurements performed on both peak and valley sites yield very similar work-function values within the large experimental uncertainty ($\phi^{\mathrm{Peak}}\mathrm{s} = 3.58 \pm 0.3$ eV, $\phi^{\mathrm{Valley}}\mathrm{s} = 3.62 \pm 0.3$ eV).

The observed peak–valley offset in FER arises from the local geometry: the pyramidal apex produces a junction field and an effective confinement which differs from the flat valley regions, which shifts the FER peak energies. This highlights that FER on corrugated surfaces is sensitive to local topography, whereas KPFS measurements show only a weak site dependence. In KPFS, the bias dependence is governed by the electrostatic force gradient [Eq. 4] and thus by the effective capacitance derivative of the full tip–sample geometry. The signal may be viewed as a superposition of a localized apex contribution and a more weakly localized background contribution from the larger tip body. On a laterally homogeneous surface, both probe the same CPD, so that tip geometry mainly affects the parabola curvature rather than the position of its minimum. On corrugated surfaces, however, the measured CPD becomes a geometry-dependent weighted average, and increasing the oscillation amplitude enhances lateral averaging and reduces the apparent peak–valley contrast.

Appendix D: Simulation model of field emission resonances

Electrostatic model of the tunneling junction

The tip apex of radius R at distance z from a planar surface is approximated by a conducting sphere with the center position at $R + z$, Fig. S6. The potential is obtained by the method of images [7]: A charge

$$q = -4\pi\varepsilon_0 R\,\frac{U}{e} \tag{S1}$$

is placed at the sphere center, and an opposite charge $q' = -q$ at $2(R+z)$ enforces the potential $V = 0$ at the surface. Higher-order images are neglected. The resulting field distribution and the field gradient along the symmetry axis

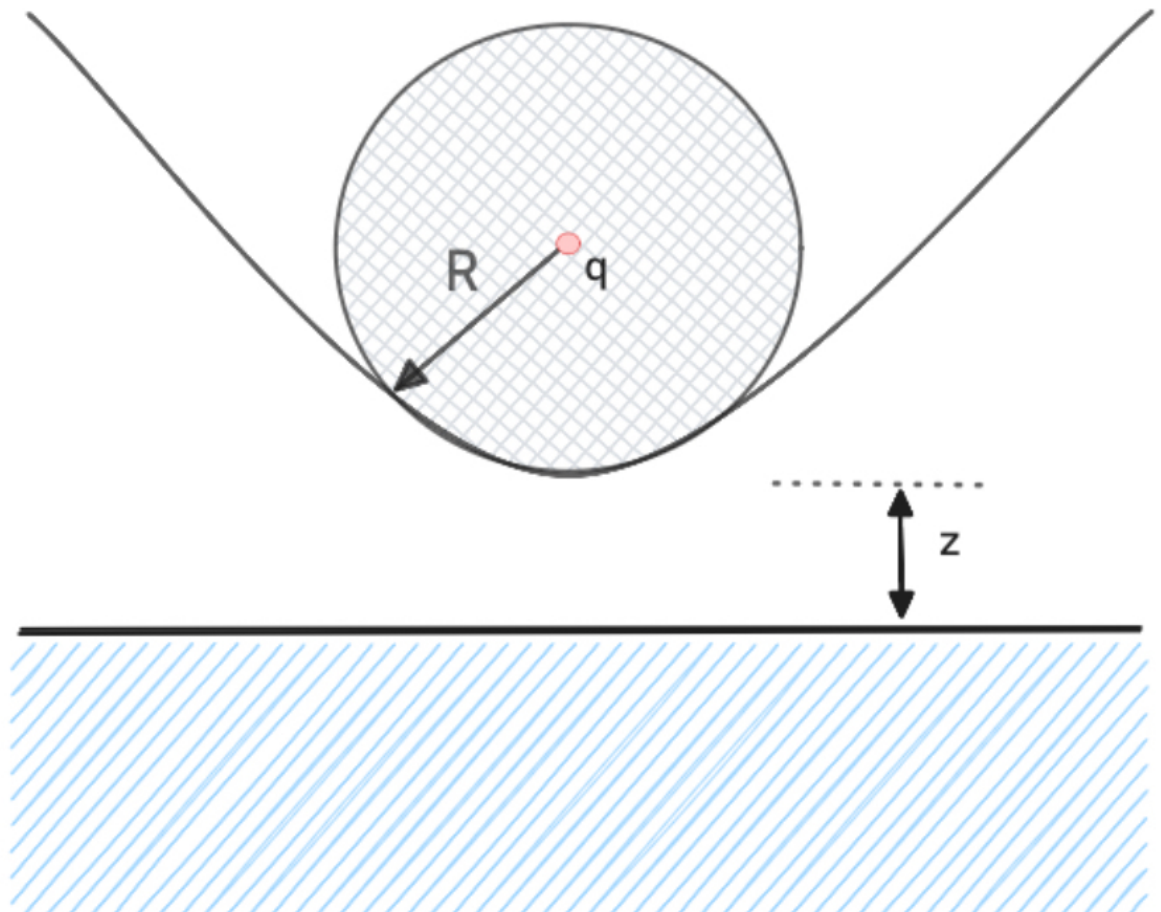

FIG. S6. Model for calculating electrical field in the tunneling junction.

depend on the R/z ratio: For $R \gg z$ the electrical field is nearly constant (plane capacitor limit), whereas for $R \sim z$ the apex field is enhanced due to geometric confinement. Thus, for $R \gtrsim 10\,\text{nm}$, the constant-field approximation is adequate, with the field gradient

$$F(z) = \frac{\phi_{\rm t} - \phi_{\rm s} + eU}{d} = \text{const.}, \tag{S2}$$

where $\phi_{\rm t}$ and $\phi_{\rm s}$ are the tip and sample work functions, respectively, U is applied bias voltage, and d is the instantaneous tip–sample distance.

In addition, the tunneling electron experiences the surface image potential [8]

$$V_{\rm im}(z) = -\frac{e^2}{16\pi\varepsilon_0 z} \qquad (z > 0), \tag{S3}$$

which in the junction adds contributions from both electrodes,

$$V_{\rm im,tot}(z) = -\frac{e^2}{16\pi\varepsilon_0}\left(\frac{1}{z} + \frac{1}{d-z}\right) + Fz. \tag{S4}$$

The divergence at $z \to 0$ is unphysical since the potential must saturate at the Fermi level $E_{\rm F}$ because occupied states block further lowering. The potential is truncated at the respective Fermi levels:

$$V(z) \to \max\bigl[V(z),\, V_{\rm min}^{\rm s,t}\bigr], \tag{S5}$$

with $V_{\rm min}^{\rm t} = -\phi_{\rm t}$ (tip side) and $V_{\rm min}^{\rm s} = -(\phi_{\rm t} + eV)$ (sample side). This ensures saturation at the Fermi energy and defines the confining potential well for field emission resonances.

Field-emission resonances: Model and fitting

We reduce the junction to a one-dimensional problem along $z \in (0, d)$ and solve

$$\left[-\frac{\hbar^2}{2m}\frac{d^2}{dz^2} + V(z; d, U)\right]\psi_n(z) = \varepsilon_n(d, U)\psi_n(z) \tag{S6}$$

on a uniform grid (finite differences). The potential combines a constant field, image terms at both electrodes, and optional dielectric corrections $\delta V_{\rm ins}(z)$ for an ultra-thin poorly conducting layer or a field enhancement factor γ for a sharp tip case. Representative potential profiles and corresponding FER spectra for different junction configurations are shown in Fig. S7.

For given values of d and U, the eigenvalues $\varepsilon_n(d, U)$ define quasi-bound levels. A FER peak appears when

$$\varepsilon_n(d, U_n) \approx 0, \tag{S7}$$

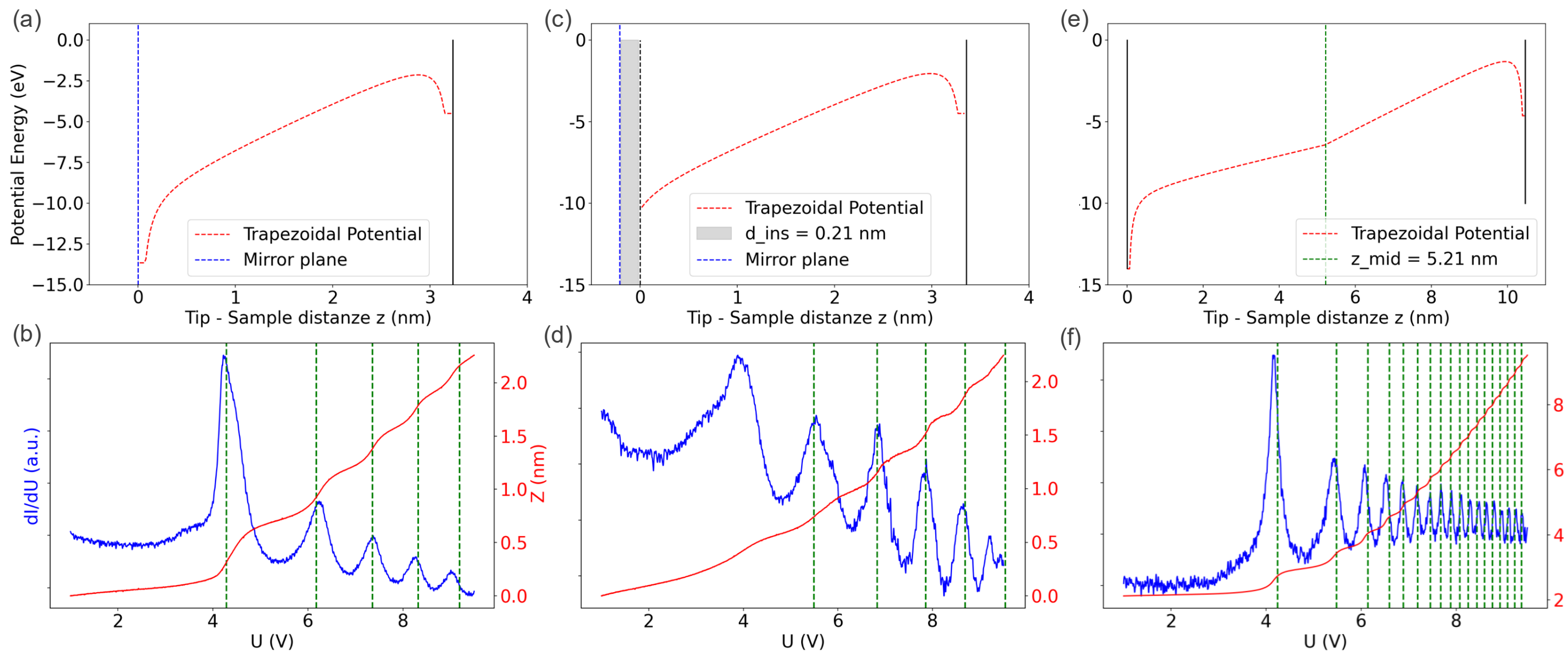

FIG. S7. (a,b) Trapezoidal potential model for a blunt metallic tip above Ag(111) together with the corresponding experimental FER spectrum (blue), tip retraction (red) and fitted resonances (green). The fitted work function of the blunt tip is $\phi_{\mathrm{t}} = 4.6\,\mathrm{eV}$ with an initial tip–sample separation $z_0 \approx 1.03\,\mathrm{nm}$; (c,d) Junction potential including a thin dielectric layer of thickness $d_{\mathrm{ins}} \approx 0.21\,\mathrm{nm}$ representing the oxidized NbO_x phase I reconstruction of the Nb(110) surface. The measured FER spectrum and simulation yield a surface work function of $\phi_{\mathrm{s}} = 4.96\,\mathrm{eV}$ and an effective initial tip–sample distance $z_0 \approx 1.22\,\mathrm{nm}$. The first peak is excluded from the simulation, as it does not origin from a FER. (e,f) Potential landscape for the same Ag(111) surface for the sharp tip obtained by controlled indentation into the Ag surface. The sharpened tip geometry introduces field enhancement, producing a steeper potential on the tip side and a shallower slope near the flat surface. The intersection of both linear potentials (indicated in the plot) defines the enhanced field region resulting from the approximation. Fitting of the FER peaks yields a tip work function of $\phi_{\mathrm{t}} = 4.9\,\mathrm{eV}$ and $z_0 \approx 3.39\,\mathrm{nm}$, with a field enhancement factor $\gamma \approx 2.3$ (as a free parameter).

i.e., when a level aligns with the tip Fermi energy. Experimentally, the peak positions $\{U_n^{\mathrm{exp}}(d_i)\}$ are recorded at tip–sample distances $d_i = d_0 + d_n$, where d_0 is the initial separation set by tunneling current and bias and d_n the incremental retractions, obtained from experiment. The optimal parameters $\Theta = \{\phi_s,\, d_0\}$ are obtained by minimizing the total squared deviation of the model from the resonance condition $\varepsilon_n = 0$.

For each measured FER at tip–sample distance d_i and bias $U_n^{\mathrm{exp}}(d_i)$ we compute the model eigenvalue $\varepsilon_n(d_i, U_n^{\mathrm{exp}}(d_i); \Theta)$, whose ideal value would be zero. The cost function is then

$$\mathcal{L}(\Theta) = \sum_{i,n} \left[\varepsilon_n\big(d_0 + d_n, U_n^{\mathrm{exp}}(d_0 + d_n); \Theta\big)\right]^2, \tag{S8}$$

which represents the sum of squared errors over all observed resonances. This accounts for field gradient, image confinement, and Fermi-level saturation and can be extended up to additional dielectric screening and tip induced field enhancement.

Dielectric layer correction

For a thin dielectric layer on top of metal surface of thickness $d_{\mathrm{ins}} \ll z$ and permittivity ε, we add a second order correction $\delta V_{\mathrm{thin}}(z)$. Physically, a thin dielectric layer partially screens the metal: The image attraction is reduced by a relative factor of the order

$$\sim \frac{\varepsilon_1}{\varepsilon_1 + \varepsilon_2} \frac{d_{\mathrm{ins}}}{z}, \tag{S9}$$

with ε_1 the permittivity of vacuum, ε_2 that of the dielectric, and z the electron–surface distance. The equation

$$\delta V_{\mathrm{thin}}(z) \simeq + \frac{e^2}{16\pi\varepsilon_0} \frac{d_{\mathrm{ins}}}{(1+\varepsilon) z^2}, \tag{S10}$$

valid for $d_{\rm ins} \ll z$. Initially we attempted to adjust the $1/z^2$ divergence at small z by a smooth cutoff approximation. However, comparison to experimental FERs showed that a more accurate description is obtained by a simple geometrical shift: The image plane on the sample side is moved from $z = 0$ to $z = -d_{\rm ins}$, i.e.

$$\frac{1}{z} \;\rightarrow\; \frac{1}{z + d_{\rm ins}}, \tag{S11}$$

which effectively accounts for the dielectric screening by referencing the image term to the underlying metallic plane. This procedure provided better agreement with experiment than artificial capping. The effect of a thin dielectric layer on the potential profile is illustrated in Fig. S7(c,d).

Extended model: Field enhancement at the tip

The standard triangular potential assumes a uniform field $F = (\phi_{\rm t} - \phi_{\rm s} + eU)/d$. However, for a sharp tip the local field is enhanced compared to the macroscopic average. Following Ref. 9, we introduce a field enhancement factor

$$\gamma = \frac{F_{\rm tip}}{F_{\rm m}}, \tag{S12}$$

where $F_{\rm m}$ is the macroscopic field in the junction near the surface when the tip is far away and $F_{\rm tip}$ the local field at the apex. In the extended model the potential profile is described by two linear gradients: A slope F on the sample side and a slope γF on the tip side. The intersection point of these lines is chosen such that the potential remains continuous. The resulting piecewise linear profile, combined with the image terms, replaces the uniform field potential in Eq. (6). The modified potential near a sharp apex and the corresponding FER behavior are depicted in Fig. S7(e,f).

Experimentally, F can be estimated from the spacing of higher-order FERs, while γ is left as a free fitting parameter. This procedure accounts for the stronger confinement near sharp tips, which increases the retraction of the tip and thus the number of observable FERs, with modified less spacing.

Appendix E: Frequency shift of all the surfaces

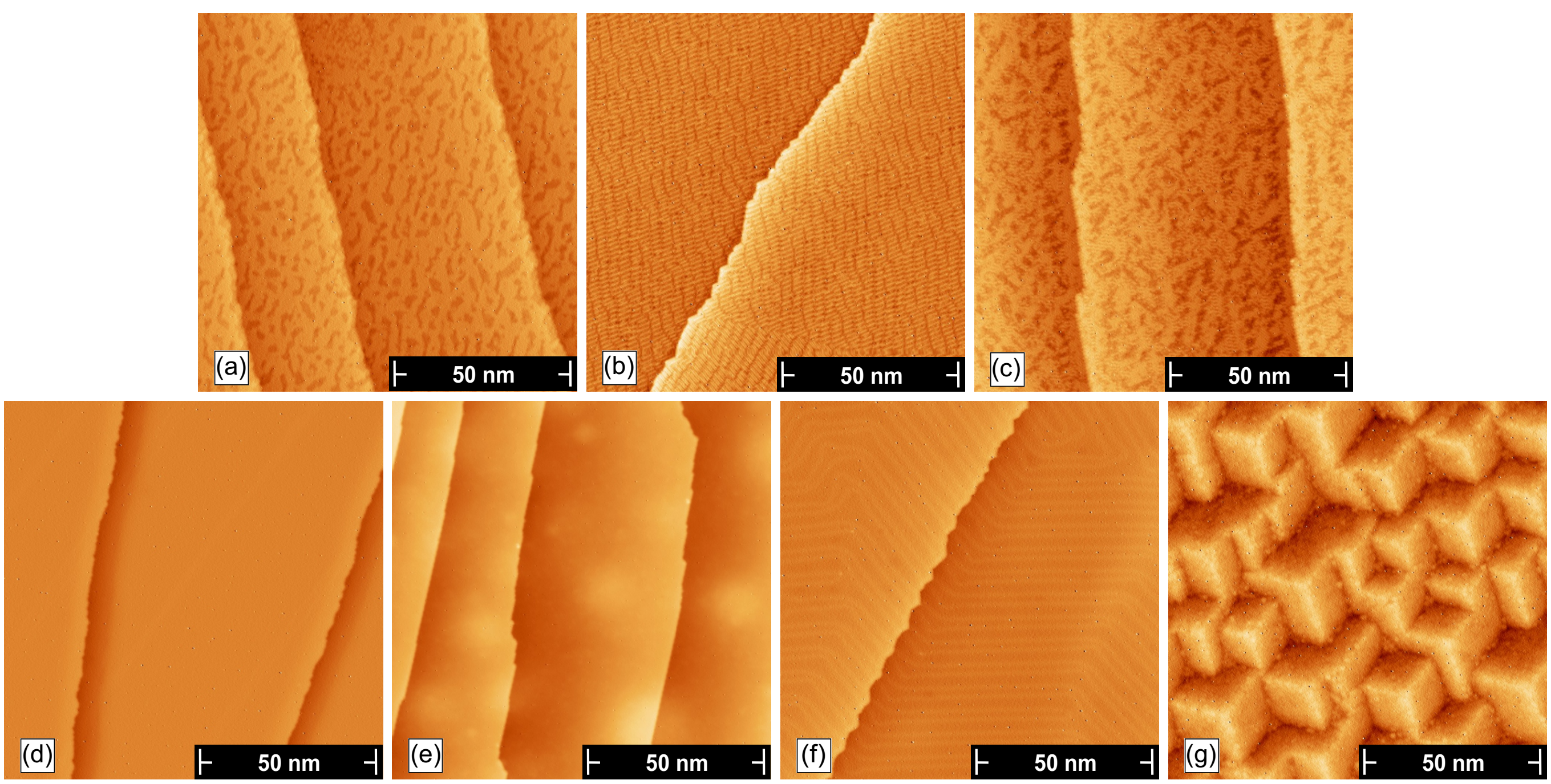

FIG. S8. Frequency shift Δf as measured by q-Plus sensor in the constant-current STM mode: (a) clean Nb(110), (b) NbO_x phase I, (c) NbO_x phase II, (d) Ag(111), (e) Ir(111), (f) Au(111), and (g) Nb(111). Imaging parameters: $z_{\rm Amp} = 7.5\,$nm; scan frame: $100\,$nm $\times$ $100\,$nm.

[1] M. Schmid, W. Hebenstreit, P. Varga, and S. Crampin, Quantum wells and electron interference phenomena in Al due to subsurface noble gas bubbles, Phys. Rev. Lett. **76**, 2298 (1996).

[2] J. V. Barth, H. Brune, G. Ertl, and R. J. Behm, Scanning tunneling microscopy observations on the reconstructed Au(111) surface: Atomic structure, long-range superstructure, rotational domains, and surface defects, Phys. Rev. B **42**, 9307 (1990).

[3] J. J. Goedecke, M. Bazarnik, and R. Wiesendanger, Stm study of Nb(111) prepared by different methods, Phys. Rev. Mater. **7**, 084803 (2023).

[4] H. Kawano, Effective work functions of the elements: Database, most probable value, previously recommended value, polycrystalline thermionic contrast, change at critical temperature, anisotropic dependence sequence, particle size dependence, Progr. Surf. Sci. **97**, 100583 (2022).

[5] K. Schouteden, B. Amin-Ahmadi, Z. Li, D. Muzychenko, D. Schryvers, and C. V. Haesendonck, Electronically decoupled stacking fault tetrahedra embedded in Au(111) films, Nature Communications **7**, 14001 (2016).

[6] E. V. Chulkov, M. Machado, and V. M. Silkin, Inverse lifetime of the surface and image states on Au(111), Vacuum **61**, 95 (2001).

[7] J. D. Jackson, *Classical electrodynamics*, 3rd ed. (Wiley, 1999).

[8] N. D. Lang and W. Kohn, Theory of metal surfaces: Work function, Phys. Rev. B **3**, 1215 (1971).

[9] W. B. Su, C. L. Lin, W. Y. Chan, S. M. Lu, and C. S. Chang, Field enhancement factors and self-focus functions manifesting in field emission resonances in scanning tunneling microscopy, Nanotechnology **27**, 175705 (2016).
